# *DNAH11* variants and its association with congenital heart disease and heterotaxy syndrome

**DOI:** 10.1038/s41598-019-43109-6

**Published:** 2019-04-30

**Authors:** Sida Liu, Weicheng Chen, Yongkun Zhan, Shuolin Li, Xiaojing Ma, Duan Ma, Wei Sheng, Guoying Huang

**Affiliations:** 1 Children’s Hospital of Fudan University, Shanghai, 201102 China; 2 Shanghai Key Laboratory of Birth Defects, Shanghai, 201102 China; 3 Key Laboratory of Molecular Medicine, Ministry of Education, Shanghai Medical College, Fudan University, Shanghai, 200000 China

**Keywords:** Cardiovascular genetics, Next-generation sequencing

## Abstract

Congenital heart diseases (CHDs) are the most common types of birth defects, affecting approximately 1% of live births and remaining the leading cause of mortality. CHD patients often show a higher incidence of heterotaxy syndrome. However, the exact aetiology of CHD and heterotaxy syndrome remains unclear. In this study, targeted sequencing and Sanger sequencing were performed to analyze the exonic regions of 37 primary ciliary dysfunction (PCD)- related candidate genes in 42 CHD patients with heterotaxy syndrome. Variants affecting protein-coding regions were filtered according to databases of known variants and predicted in silico using functional prediction program. Thirty-four potential disease-causing heterozygous variants in 11 genes were identified in the 19 CHD patients with heterotaxy syndrome (45.2%, 19/42). The *DNAH11* gene showed the highest mutation rate (16.7%; 14 of 84 alleles) among the CHD patients with heterotaxy. Fisher’s exact test revealed a significant association of *DNAH11* variants with CHD and heterotaxy (*P* = 0.0001). In families, six different compound heterozygous variants of *DNAH11* were validated in family 1-5031 (p.W802X/p.M282I), family 2-5045 (p.T3460K/p.G4425S), family 3-5065 (p.G447R/p.L1157R), family 4-5130 (p.I2262T/p.D3800H), family 5-5707 (p.S1823fs/p.F2759L/p.R4395X) and family 6-5062 (p.D3610V/p.I243V). These findings suggest that the *DNAH11* variants are significantly associated with CHD and heterotaxy syndrome and that compound heterozygous *DNAH11* variants may be the common genetic cause of the development of familial CHD and heterotaxy syndrome.

## Introduction

Congenital heart diseases (CHDs) are the most common types of birth defects, affecting approximately 1% of live births and remaining the leading cause of mortality^1^. Interestingly, CHD patients often show a higher incidence of heterotaxy syndrome. Studies have found that approximately 5–10% of CHD patients present with heterotaxy syndrome^2^. Heterotaxy(HTX) is a rare birth defect involving left-right (LR) asymmetry with an incidence of 1 in 10,000 newborns, and approximately 90% of HTX patients have complex CHDs^3^. CHD and heterotaxy syndrome have been shown to be associated with primary cilia dysfunction (PCD) or cilia dysfunction (CD). The mortality and respiratory complications of CHD and heterotaxy in patients after cardiac surgery are significantly higher than those in the same type of patients without heterotaxy^4^. However, PCD is considered to be a monogenic heterogeneous recessive disorder, while CHD and heterotaxy syndrome are multiple-gene complex inherited diseases^5^. The linkage between PCD and CHD /HTX needs to be investigated.

Studies have shown that mutations in genes causing PCD may be associated with the development of heterotaxy and/or CHD syndrome. Approximately 50% of PCD patients exhibit heterotaxy associated with complex CHDs^6^. PCD-related genes in heterotaxy are thought to be responsible for the function of motile cilia in LR patterning, and CHD/heterotaxy patients also show increased airway CD similar to that seen in PCD patients^7^.

Researchers have shown that CHD and heterotaxy syndromes are multiple, complex, inherited diseases caused by numerous genes that are responsible for inherited and sporadic cases^8^. Mutations in over 15 genes related to LR patterning have been observed in heterotaxy patients, but these findings account for fewer than 20% of heterotaxy cases^9^. You Li *et al*. performed whole-exome sequencing in 218 CHD mouse models and identified 91 recessive CHD mutations in 61 genes, including 34 cilia-related genes, 16 genes involved in cilia-transduced cell signalling, and 10 genes regulating vesicular trafficking (a pathway important for ciliogenesis and cell signaling), including a loss-of-function *DNAH11* mutation known to cause PCD^10^.

*DNAH11*, located on chromosome 7p15.3, encodes a ciliary outer dynein arm protein (520 kDa) and is a member of the dynein heavy chain family, localizing exclusively to the proximal region of respiratory cilia^11^. *DNAH11* mutations have been found to result in abnormal ciliary ultrastructure and hyperkinetic ciliary beating^12^. Gene editing of *DNAH11* mutations can restore normal cilia motility in primary ciliary dyskinesia^13^. An overwhelming majority of previous studies related to *DNAH11* have focused on PCD and situs inversus totalis, and few studies have concentrated on heterotaxy and CHD.

To date, over 40 PCD-related genes have been found in heterotaxic patients with PCD. However, whether PCD-related gene mutations are associated with CHD and heterotaxy syndrome remains unclear. In this study, we performed targeted sequencing and Sanger sequencing analysis of PCD-related genes in Chinese CHD patients with heterotaxy syndrome to explore the genetic aetiology of CHD and heterotaxy syndrome.

## Results

### Assessment of the ciliary movement status in CHD patients with heterotaxy syndrome

A cohort of 42 CHD patients with heterotaxy syndrome were recruited from unrelated families, including 14 females (33.3%) and 28 males (66.7%) with ages ranging from 0.1 to 20.1 years (mean ± SD: 5.6 ± 4.7 years). To assess the ciliary movement status, we examined the ciliary beat pattern using a slow-motion playback of the video sequence to generate tracings of the ciliary beat and found that 29 cases (69%, 29/42) showed CD (18 males and 11 females), and 13 cases (31%, 13/42) presented normal ciliary function (10 males and 3 females).

The clinical features of the study subjects are summarized in Table 1.

**Table 1:** Clinical characteristics of the study subjects.

Characteristic	CHD/heterotaxy + CD^a^	CHD/heterotaxy + no CD^b^	CHD/heterotaxy (Total)
Patients (%)	29 (69.1%)	13 (30.9%)	42 (100%)
Age (mean ± SD)	5.4 ± 5.0 (years)	6 ± 4.4 (years)	5.6 ± 4.7 (years)
Male (%)	18 (42.9)	10 (23.8)	28 (66.7%)
Female (%)	11 (26.2)	3 (7.1)	14 (33.3%)

^a^CHD/heterotaxy + CD: congenital heart disease/heterotaxy with ciliary dysfunction.

^b^CHD/heterotaxy + CD: congenital heart disease/heterotaxy without ciliary dysfunction.

### Targeted sequencing analysis of gene variants in CHD patients with heterotaxy syndrome

To investigate the role of gene variants in CHD/heterotaxy diseases, we carried out targeted sequencing on the exonic regions of the following 37 PCD-related candidate genes in 42 CHD patients with heterotaxy: *ABCC4*, *ARMC4*, *C21orf59*, *CCDC39*, *CCDC40*, *CCDC65*, *CCDC114*, *CCDC151*, *CCNO*, *DNAAF1*, *DNAAF2*, *DNAFF3*, *DNAAF5*, *DNAH5*, *DNAH8*, *DNAH11*, *DNAI1*, *DNAI2*, *DNAL1*, *DRC1*, *DYX1C1*, *HEATR2*, *HYDIN*, *LRRC6*, *NAT10*, *NME8*, *PTGES*, *PTGES2*, *PTGES3*, *PTGER4*, *PTGS1*, *PTGS2*, *RSPH1*, *RSPH4A*, *RSPH9*, *SPAG1* and *ZMYND10*.

For these genes, 758 regions of gene coding exons and their intron/exon junctions were sequenced. The coverage of the sequencing results was 90%-99%, with an average coverage of approximately 97%, and the average depth of sequencing was 100X. By filtering from 1000 Genomes Project and ExAC databases and using SIFT, PolyPhen2 and MutationTaster prediction programs, we focused on novel or rare coding variants (MAF < 0.01%) present in CHD patients with heterotaxy, excluding the common variants, synonymous variants and non-synonymous variants that are predicted to have no deleterious effect on protein function.

Thirty-four potential disease-causing heterozygous variants were identified in 11 of 37 candidate genes, including *ARMC4*, *CCDC40*, *CCDC65*, *DHAH8*, *DNAAF2*, *DNAH11*, *DNAH5*, *DNAH8*, *DRC1*, *HYDIN*, and *SPAG1* (Table 2). These mutated genes were distributed in 19 CHD/HTX patients (45.2%, 19/42). Eighteen CHD patients with heterotaxy and CD had 31 variants in 8 genes, and 1 CHD patient (patient #5030) with heterotaxy and normal ciliary function possessed 3 variants in 3 genes. These findings indicate that the CHD patients with heterotaxy and CD has a significantly higher gene mutation rate than the CHD patients with heterotaxy and normal ciliary function among the CHD patients with heterotaxy (62.1%, 18/29 vs. 7.7%, 1/13; *P* = 0.0018) (Table 3).

**Table 2:** The gene variants detected in CHD patients with heterotaxy.

Patient number	Patient	Gender	Age (year)	Ciliary motion pattern*	Gene	Base change	Amino acid change
1	#5031	M	3.8	I + R + D	*DNAH11*	c.G2406A	p.W802X
					*DNAH11*	c.G846C	p.M282I
2	#5045	M	4.4	R + D	*DNAH11*	c.C10379A	p.T3460K
					*DNAH11*	c.G13273A	p.G4425S
3	#5065	M	5.4	I + R	*DNAH11*	c.G1339A	p.G447R
					*DNAH11*	c.T3470G	p.L1157R
4	#5130	F	3.6	R + D	*DNAH11*	c.T6785C	p.I2262T
					*DNAH11*	c.G11398C	p.D3800H
					*CCDC65*	c.A881G	p.K294R
					*DNAAF2*	c.C1753T	p.P585S
5	#5707	M	0.5	R + I	*DNAH11*	c.5470dupC	p.S1823fs
					*DNAH11*	c.T8275C	p.F2759L
					*DNAH11*	C13183T	p.R4395X
6	#5062	F	8.6	I + R	*DNAH11*	c.A10829T	p.D3610V
					*DNAH11*	c.A727G	p.I243V
					*HYDIN*	c.C2503T	p.H835Y
7	5033	M	6.8	R + D	*DNAH11*	c.C6983T	p.P2328L
					*HYDIN*	c.A9022C	p.N3008H
8	5176	F	1.5	R + D	*HYDIN*	c.C7492T	p.R2498C
					*DNAH8*	c.A12517G	p.M4173V
9	5071	M	5.7	R + D	*DNAH8*	c.C4690T	p.P1564S
					*ARMC4*	c.C1679T	p.A560V
10	5040	M	3.2	R + D + I	*DNAH8*	c.G6730A	p.V2244I
11	5145	F	3.7	R	*DHAH8*	c.C4690T	p.P1564S
12	5043	M	2.2	I	*DNAH5*	c.A3086T	p.D1029V
13	5063	M	4.3	none	*DNAH5*	c.A10169G	p.D3390G
14	5032	F	16.7	R + D	*DNAH5*	c.G13364T	p.G4455V
15	5053	M	4.2	R + D	*DNAH5*	c.G12212A	p.R4071H
16	5055	M	1.2	none	*HYDIN*	c.G3252A	p.V1085M
17	5133	F	4.5	none	*HYDIN*	c.C8905T	p.R2969W
18	5064	M	19.2	R + D	*CCDC40*	c.C1669T	p.R557W
19	5030	F	13.3	N	*DNAH8*	c.G12721A	p. A4241T
					*SPAG1*	c.G225T	p.L75F
					*DRC1*	c. G350A	p. R117H
20	5076	F	11.7	N	none	none	none
21	5056	M	3.9	N	none	none	none
22	5088	M	8.2	N	none	none	none
23	5066	F	4.4	R + D	none	none	none
24	5087	F	8.8	R + D	none	none	none
25	5041	M	3	R + D	none	none	none
26	5044	F	3	R + D	none	none	none
27	5048	M	3	R + D	none	none	none
28	5029	M	5.3	N	none	none	none
29	5035	M	7.5	N	none	none	none
30	5049	M	1.4	N	none	none	none
31	5155	M	3.9	N	none	none	none
32	5072	M	4.9	N	none	none	none
33	5078	M	4.6	N	none	none	none
34	5101	M	0.1	N	none	none	none
35	5050	M	4.2	R + D	none	none	none
36	5052	F	20.1	R + D + I	none	none	none
37	5061	M	1.3	R + D	none	none	none
38	5020	M	1.9	none	none	none	none
39	5191	F	4	R + D	none	none	none
40	51531	M	4.5	W	none	none	none
41	51557	M	0.8	N	none	none	none
42	5037	F	12.4	N	none	none	none

^*^I: immotile; D: discordance; W: wave; R: restricted; None: no cilia; N: normal.

**Table 3:** The gene variant rate between CHD/heterotaxy patients with ciliary dysfunction and CHD/heterotaxy patients without ciliary dysfunction.

Characteristic	CHD/heterotaxy + CD^A^ (n = 29)	CHD/heterotaxy + no CD^B^ (n = 13)	*P* ^a^
Gene mutation rate (%)	18 (62.1%)	1 (7.7%)	0.0018

^A^CHD/heterotaxy + CD: congenital heart disease/heterotaxy with ciliary dysfunction.

^B^CHD/heterotaxy + no CD: congenital heart disease/heterotaxy without ciliary dysfunction.

^a^Fisher’s exact test was used.

### Association of *DNAH11* variants with the risk of CHD and heterotaxy syndrome

As shown in Table 4, the *DNAH11* gene showed the highest mutation rate (16.7%; 14 of 84 alleles) among the CHD patients with heterotaxy. The *HYDIN* and *DNAH8* genes both showed 6% mutation rate (5 of 84 alleles). The mutation rate of *DNAH5* genes was 4.8% (4 of 84 alleles), and the other genes (*ARMC4*, *CCDC40*, *CCDC65*, *DHAH8*, *DNAAF2*, *DRC1*, and *SPAG1*) all showed a 1.2% mutation rate (1 of 84 alleles).

**Table 4:** The variant frequency of the detected genes in CHD patients with heterotaxy.

Gene	Mutation type	Base change	Amino acid change	Cases with variants	Frequency (%)
***DNAH11***	**Stop-gain**	**c**.**G2406A**	**p**.**W802X**	**5031**	**14/84** (**16**.**7%**)
	Missense	c.G846C	p.M282I	5031	
	Missense	c.C10379A	p.T3460K	5045	
	Missense	c.G13273A	p.G4425S	5045	
	Missense	c.G1339A	p.G447R	5065	
	Missense	c.T3470G	p.L1157R	5065	
	Missense	c.T6785C	p.I2262T	5130	
	Missense	c.G11398C	p.D3800H	5130	
	Frameshift	c.5470dupC	p.S1823fs	5707	
	Missense	c.T8275C	p.F2759L	5707	
	Stop-gain	C13183T	p.R4395X	5707	
	Missense	c.A10829T	p.D3610V	5062	
	Missense	c.A727G	p.I243V	5062	
	Missense	c.C6983T	p.P2328L	5033	
***HYDIN***	**Missense**	**c**.**A9022C**	**p**.**N3008H**	**5033**	**5/84** (**6%**)
	Missense	c.G3252A	p.V1085M	5055	
	Missense	c.C2503T	p.H835Y	5062	
	Missense	c.C8905T	p.R2969W	5133	
	Missense	c.C7492T	p.R2498C	5176	
***DNAH8***	**Missense**	**c**.**G12721A**	**p**. **A4241T**	**5030**	**5/84** (**6%**)
	Missense	c.G6730A	p.V2244I	5040	
	Missense	c.C4690T	p.P1564S	5071, 5145	
	Missense	c.A12517G	p.M4173V	5176	
***DNAH5***	**Missense**	**c**.**G13364T**	**p**.**G4455V**	**5032**	**4/84** (**4**.**8%**)
	Missense	c.A3086T	p.D1029V	5043	
	Missense	c.G12212A	p.R4071H	5053	
	Missense	c.A10169G	p.D3390G	5063	
*SPAG1*	Missense	c.G225T	p.L75F	5030	1/84 (1.2%)
*ARMC4*	Missense	c.C1679T	p.A560V	5071	1/84 (1.2%)
*CCDC40*	Missense	c.C1669T	p.R557W	5064	1/84 (1.2%)
*CCDC65*	Missense	c.A881G	p.K294R	5130	1/84 (1.2%)
*DHAH8*	Missense	c.C4690T	p.P1564S	5145	1/84 (1.2%)
*DNAAF2*	Missense	c.C1753T	p.P585S	5130	1/84 (1.2%)
*DRC1*	Missense	c. G350A	p. R117H	5030	1/84 (1.2%)

A total of 34 variants in 11 genes.

Concerning the highest mutation rate in the *DNAH11* gene, we further analyzed the association of *DNAH11* mutations with CHD and heterotaxy syndrome. In this study, there were 14 mutations in the *DNAH11* genes in 7 of 42 patients, including 11 missense, 1 frameshift and 2 stop-gain mutations. Eight of these variants were novel and not present in the ExAC or 1000 Genomes Project databases, and 6 were low-frequency variants (MAF < 0.01%). Conservation analysis was processed via UCSC Genome Browser hg19.

In our previous study, whole genome sequencing was performed in 98 CHD patients without heterotaxy, and the methodology used in the previous study is strictly the same as that used in the present investigation. The CHD subtypes of these 98 CHD patients are listed in Table 5.

**Table 5:** The CHD subtypes of 98 CHD patients.

CHD subtypes	Number of Cases
Congenital heart defects. total cases	98
Single ventricle, total	3
DIRV, DILV	3
Single ventricle indeterminate, unspecified	0
Conotruncal	18
Truncus arteriosus	0
TOF	13
D-loop TGA (includes TGA with VSD)	1
DORV	4
L-loop TGA, not single ventricle	0
AVCD (AVSD), complete	8
AVCD (AVSD), partial	7
Left-sided obstructive defects	3
Aortic stenosis	1
Coarctation	2
Hypoplastic left heart syndrome	0
Right-sided defects	18
DCRV/anomalous muscle bundle of the right ventricle	5
Ebstein anomaly	0
Pulmonary stenosis	6
Pulmonary atresia with intact septum	1
Non-TOF pulmonary atresia with VSD	6
TAPVD	2
PAVPD	1
Ventricular septal defects, total	34
VSD membranous	31
VSD malalignment-type	3
Atrial septal defects, secundum	24
Dysplasia or absent right valve	2
Dysplasia or absent left valve	1
PDA	11
SVC/persistent left or bilateral	8
ALCAPA	1
Cor triatriatum	2
APW	1

ALCAPA, anomalous left coronary artery originating from the pulmonary artery; APW, aortopulmonary window; ASD, atrial septal defect; AVCD (AVSD), atrioventricular canal defect (atrioventricular septal defect); DCRV, double chambered right ventricle; DIRV, DILV, double inlet right ventricle, double inlet left ventricle; DORV, Double outlet right Ventricle; PDA, patent ductus arteriosus; SVC, superior vena cava; T/PAPVR, totally/partially anomalous pulmonary venous return; TGA, transposition of the great arteries; TOF, tetralogy of Fallot; VSD, ventricular septal defect.

By consulting the previous exome database in our laboratory, we found no disease-causing mutations in the *DNAH11* gene among 98 CHD cases. Moreover, one *DNAH11* mutation (c.A9584G:p.N3195S) was found in 1 case (Patient 2073) among 3 CHD patients with heterotaxy. Combined with the results showing 7 of 42 patients with *DNAH11* mutations in this study, the prevalence of *DNAH11* mutations was higher in CHD patients with heterotaxy (8 of 45 cases) than in CHD patients (0 of 98 cases). The 98 CHD cases were considered controls because these cases exhibited only CHD, and the association of *DNAH11* mutations with CHD and heterotaxy was significant (8 of 45 CHD patients with heterotaxy vs. 0 of 98 controls, *P* = 0.0001 by Fisher’s exact test). These findings suggest a significant association of *DNAH11* mutations with the risk of CHD and heterotaxy syndrome (Table 6).

**Table 6:** Association of *DNAH11* mutations with the risk of congenital heart disease and heterotaxy syndrome.

Characteristic	CHD/heterotaxy cases^a^ (n = 45)	CHD cases (n = 98)	*P* ^b^
No. of patients with mutation	8	0	0.0001

^a^CHD/heterotaxy: congenital heart disease/heterotaxy; ^b^Fisher’s exact test was used.

### *DNAH11* compound heterozygous mutations in CHD families with heterotaxy

Disease-causing *DNAH11* mutations are inherited by autosomal recessive inheritance. Because there were no homozygous mutations in *DNAH11* in this study, we focused on compound heterozygous mutations in the *DNAH11* gene, which may be the main cause of the development of CHD/heterotaxy. The 14 disease-causing heterozygous mutations in *DNAH11* were distributed among 7 CHD patients with heterotaxy, with 6 patients having two or more *DNAH11* mutations. These *DNAH11* mutations were further confirmed to be present in the available DNA of parents and other family members of the patients by Sanger sequencing. Interestingly, six different compound heterozygous variants in *DNAH11* were validated respectively in six different families, including family 1-5031 (p.W802X/p.M282I), family 2-5045 (p.T3460K/p.G4425S), family 3-5065 (p.G447R/p.L1157R), family 4-5130 (p.I2262T/p.D3800H), family 5-5707 (p.S1823fs/p.F2759L/p.R4395X) and family 6-5062 (p.D3610V/p.I243V) (Table 7).

**Table 7:** The compound heterozygous variants in the *DNAH11* gene in CHD families with heterotaxy syndrome.

Family number	Proband	Gene	Base change	Amino acid change	Mutation type	SIFT^a^	Polyphen2^a^	Mutation Taster^a^	ExAC^b^	1000_Genomes Project^b^
1	#5031	*DNAH11*	c.G2406A	p.W802X	Stop-gain	D	NA	A	0	0
		*DNAH11*	c.G846C	p.M282I	Missense	D	B	N	<0.001	<0.001
2	#5045	*DNAH11*	c.C10379A	p.T3460K	Missense	D	D	D	<0.001	<0.001
		*DNAH11*	c.G13273A	p.G4425S	Missense	D	D	D	0	0
3	#5065	*DNAH11*	c.G1339A	p.G447R	Missense	T	B	N	0	0
		*DNAH11*	c.T3470G	p.L1157R	Missense	D	D	D	<0.001	<0.001
4	#5130	*DNAH11*	c.T6785C	p.I2262T	Missense	D	D	D	0	0
		*DNAH11*	c.G11398C	p.D3800H	Missense	T	B	D	0	0
5	#5707	*DNAH11*	c.T8275C	p.F2759L	Missense	T	D	D	<0.001	<0.001
		*DNAH11*	C13183T	p.R4395X	Stop-gain	NA	NA	A	NA	0
		*DNAH11*	c. 5470dupC	S1823fs	Frameshift	NA	NA	NA	NA	NA
6	#5062	*DNAH11*	c.A10829T	p.D3610V	Missense	T	D	D	<0.001	<0.001
		*DNAH11*	c.A727G	p.I243V	Missense	T	D	D	<0.001	<0.001

^a^Mutation assessment by SIFT, PolyPhen-2 (PPH2) and Mutation Taster. T: tolerant; P: probably damaging; D: disease causing; B: Benign; A: disease causing automatic; N: polymorphism.

^b^Frequency of corresponding mutations in all populations of the ExAC Browser and 1000 Genomes Project (1KG). NA, not available.

**Figure 1:**
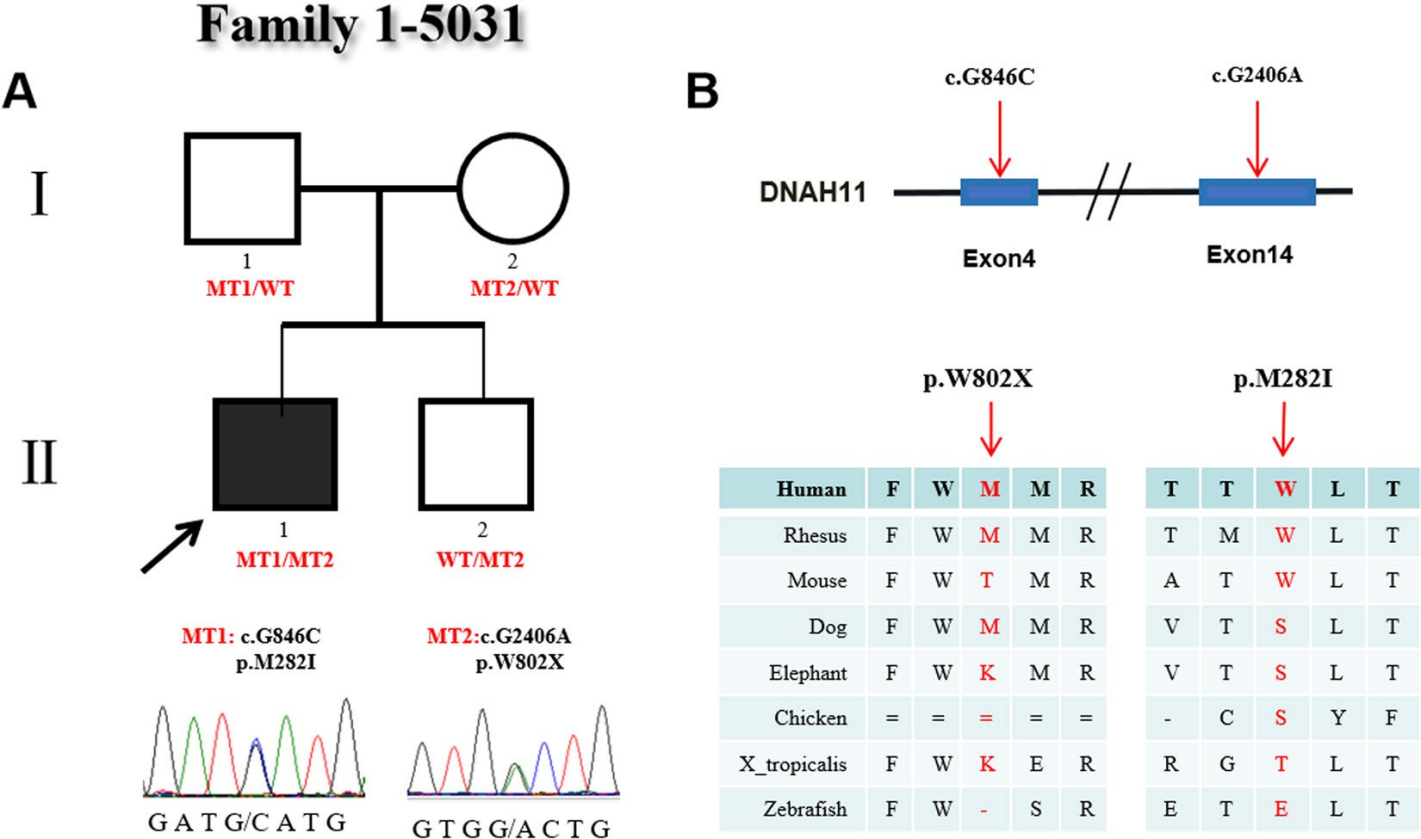
Identification of mutations in *DNAH11* in family 1. (**A**) The pedigree of family 1 (#5031). The proband from family 1 has two heterozygous mutations in *DNAH11*. The other members in this family are carriers. Sanger sequencing confirmation is shown below the pedigrees. A square represents male and a circle represents female. A black arrow indicates the proband. (**B**) Locations and conservation of mutations in *DNAH11*. The positions of mutations are indicated in the genomic structure of *DNAH11*. The amino acid changes were compared among eight mammalian species by conservation analysis. MT1: c.G846C (p.M282I); MT2: c.G2406A (p.W802X).

In family 1 (Fig. 1A), there were four members. The proband (#5031), who carried 2 heterozygous mutations (c.G2406A:p.W802X and c.G846C:p.M282I), was male and diagnosed with CHD and heterotaxy, including isolated right heart, complete atrioventricular canal (CAVC), double outlet right ventricle (DORV) and atrial septal defect (ASD), and showed abnormal ciliary function. His parents and younger brother were all without clinical manifestations, but their ciliary movements were abnormal as evidenced by uncoordinated ciliary waves. In this family, the heterozygous variant c.G2406A (p.W802X) was a *novo* stop-gain mutation located on exon 14 of *DNAH11*. This variant was present in the proband and his young brother and inherited from their mother. Functional analysis showed the variant p.W802X was predicted to be damaging by SIFT software and conserved among human, rhesus and dog species. The other heterozygous variant c.G846C (p.M282I) located in exon 4 in the proband was transmitted from his father and was not observed in his young brother and mother. This mutation was reported in the Exome Aggregation Consortium (ExAc) (0.00006547) and 1000 Genomes Project (0.000199681) databases. The p.M282I change was also predicted to be damaging by SIFT software and conserved among human, rhesus and mouse species (Fig. 1B).

**Figure 2:**
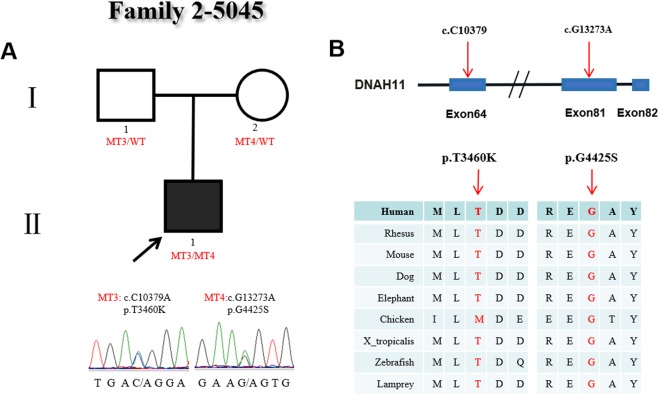
Identification of mutations in *DNAH11* in family 2. (**A**) The pedigree of family 2 (#5045). The proband has two heterozygous mutations in *DNAH11*. The other members in this family are carriers. Sanger sequencing confirmation is shown below the pedigrees.A square represents a male, and a circle represents a female. A black arrow indicates the proband. (**B**) Locations and conservation of mutations in *DNAH11*. The positions of the mutations are indicated in the genomic structure of *DNAH11*. The amino acid changes were compared among eight mammalian species by conservation analysis. MT3: c.C10379A (p.T3460K), MT4: c.G13273A (p.G4425S).

In family 2, the proband (#5045) was male and diagnosed with CHD and heterotaxy, showing abnormal ciliary movement. His parents were both heterozygous carriers and showed normal phenotypes. The heterozygous variants (c.C10379A:p.T3460K/c.G13273A:p.G4425S) of the *DNAH11* gene were confirmed in family 2 by Sanger sequencing (Fig. 2A). Of the two heterozygous variants, c.C10379A (p.T3460K) was a missense variant type located in exon 64 and was inherited from the proband’s mother and presented in the ExAc (0.0001879) and 1000 Genomes Project (0.000199681) databases. The amino acid p.T3460K alteration is predicted to be damaging by SIFT, PolyPhen2 and MutationTaster and is highly conserved among mammalian species but not in chickens. The other variant, c.G13273A (p.G4425S), was a novel missense variant type located in exon 81 and which was transmitted from the proband’s carrier father. The p.G4425S change was predicted to be damaging by the SIFT, PolyPhen2 and MutationTaster software programs and is highly conserved among mammalian species (Fig. 2B).

**Figure 3:**
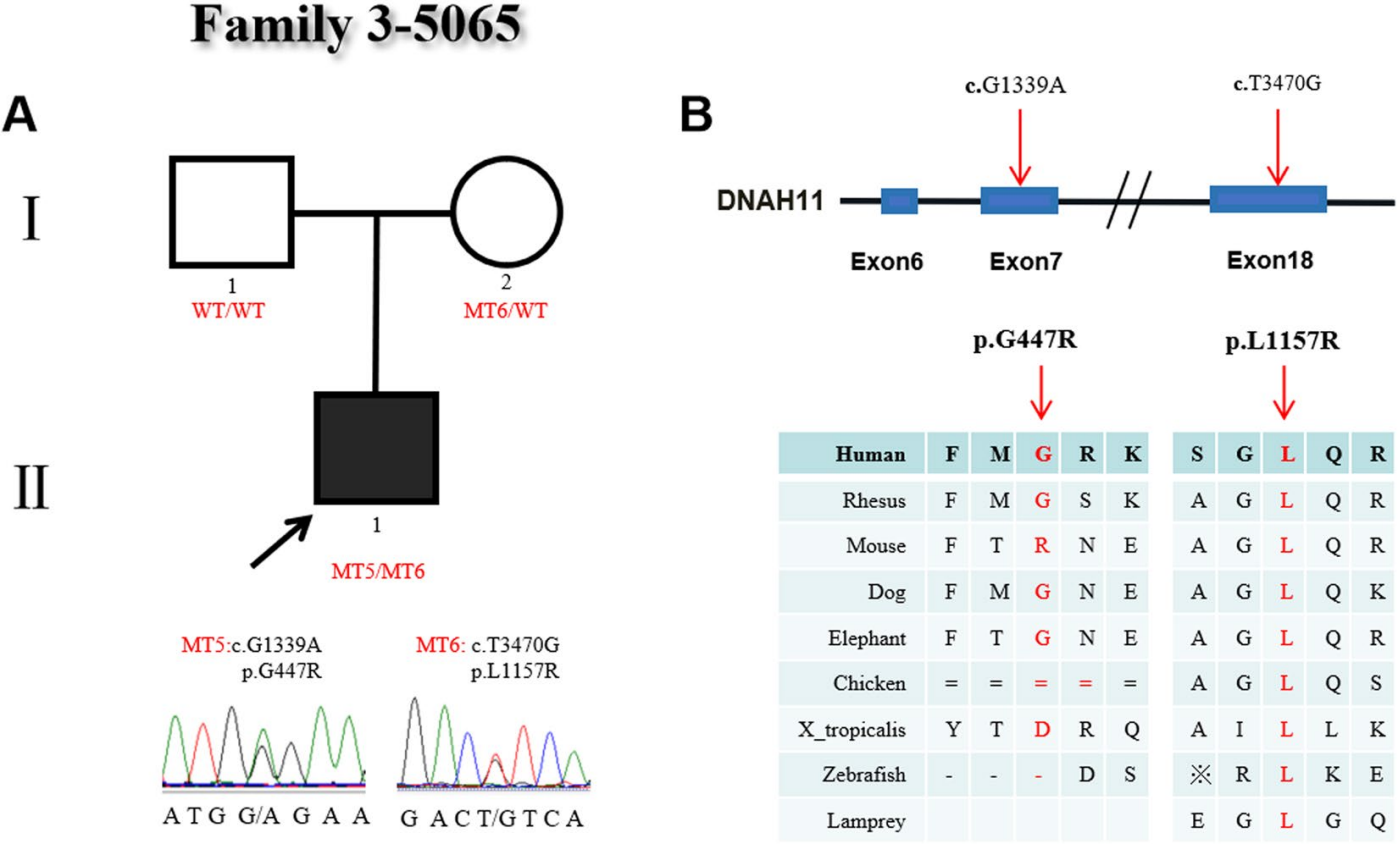
Identification of mutations in *DNAH11* in family 3. (**A**) The pedigree of family 3 (#5065). The proband carries two heterozygous mutations in *DNAH11*. The proband’s mother is a carrier, and his father shows no mutation in *DNAH11*. Sanger sequencing confirmation is shown below the pedigrees. A square represents male and a circle represents female. A black arrow indicates the proband. (**B**) Locations and conservation of mutations in *DNAH11*. The positions of mutations are indicated in the genomic structure of *DNAH11*. The amino acid changes were compared among eight mammalian species by conservation analysis. MT5: c.G1339A (p.G447R); MT6: c.T3470G (p.L1157R).

The family 3 proband (#5065) had severe CHD and heterotaxy, with abnormal ciliary movement. His parents were normal and healthy. This family carried two heterozygous missense mutations (c.G1339A:p.G447R/c.T3470G:p.L1157R) in the proband (Fig. 3A). The c.G1339A (p.G447R) in exon 7 was a novel variant and absent in his parents, indicating that this variant was *de novo*. The altered amino acid p.G447R was conserved among the human, rhesus, dog and elephant species. Functional analysis of this change was predicted to be benign by prediction software. The other c.T3470G (p.L1157R) in exon 18 was a missense mutation type and presented in the ExAc (0.000149) and 1000 Genomes Project (0.000199) databases. The mutation c.T3470G was inherited from his mother, resulting in the substitution of the 1157 amino acid Leu (L) with Arg (R) (p.L1157R) and was predicted to be a damaging change in the protein. The altered amino acid p.L1157R is highly conserved among many species. Although the p.G447R variant was predicted to be not damaging, considering the proband’s mother normal phenotype, we concluded that the combined effects of the two heterozygous variants (p.G447R/p.L1157R) may be an important factor causing CHD/heterotaxy disease (Fig. 3B).

**Figure 4:**
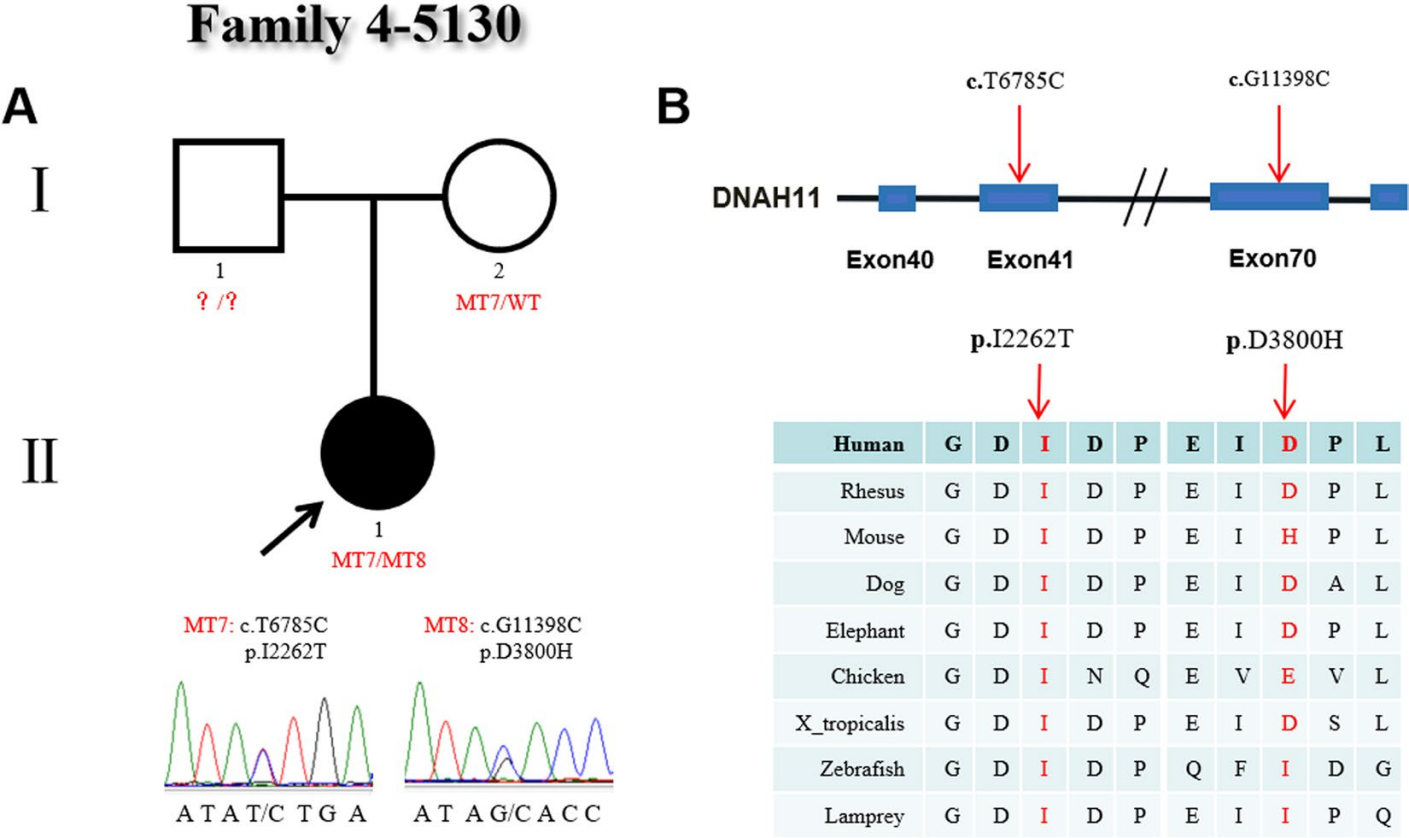
Identification of mutations in *DNAH11* in family 4. (**A**) The pedigree of family 4 (#5130). The proband has two heterozygous mutations in *DNAH11*. The proband’s mother is a carrier, and the blood sample of his father was not obtained. Sanger sequencing confirmation is shown below the pedigrees. A square represents a male and a circle represents a female. A black arrow indicates the proband. (**B**) Locations and conservation of mutations in *DNAH11*. The positions of mutations are indicated in the genomic structure of *DNAH11*. The amino acid changes were compared among eight mammalian species by conservation analysis. MT7: c.T6785C (p.I2262T); MT8: c.G11398C (p.D3800H).

The family 4 proband (#5130), who was female and diagnosed with CHD, heterotaxy and CD, carried two heterozygous missense variants (c.T6785C:p.I2262T/c.G11398C:p.D3800H). We obtained the proband and her mother’s blood sample for validating the detected variants by Sanger sequencing. The blood sample of the proband’s father was not obtained. However, the parents both had normal phenotypes according to the medical history record (Fig. 4A). The c.T6785C (p.I2262T) variant was a novel heterozygous type and was located in exon 41 of *DNAH11*, which was inherited from the proband’s mother. Functional analysis indicated that the p.I2262T variant was predicted to be damaging by the SIFT, PolyPhen2 and Mutation Taster software programs and was highly conserved among many species. The variant c.G11398C (p.D3800H) in exon 70 was also a *novel* heterozygous variant and was absent from the ExAc and 1000 Genomes Project databases. Functional analysis predicted that the variant p.D3800H was damaging (by MutationTaster software) and was conserved among different species (Fig. 4B). We could not collect a blood sample from the proband’s father; therefore the inherited origin of the c.G11398C (p.D3800H) variant could not be determined, and we proposed that the compound heterozygous variants (p.I2262T/p.D3800H) may be associated with the development of the proband’s disease.

**Figure 5:**
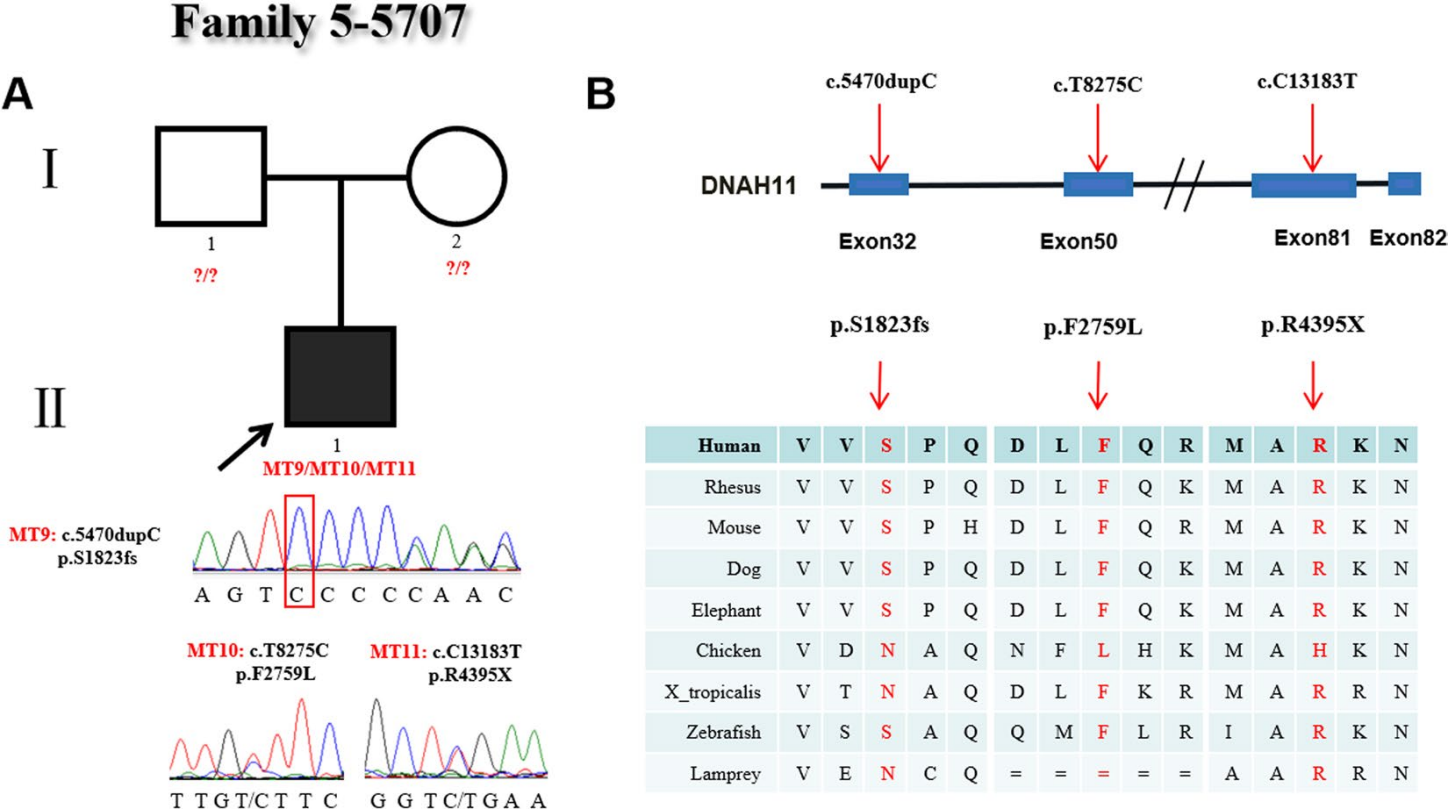
Identification of mutations in *DNAH11* in family 5. (**A**) The pedigree of family 5 (#5707). The proband carries one frameshift insertion and two heterozygous mutations in *DNAH11*. The blood samples of the proband’s parents were not obtained. Sanger sequencing confirmation is shown below the pedigrees.A square represents a male, and a circle represents a female. A black arrow indicates the proband. A red square indicates the “dupC”. (**B**) Locations and conservation of mutations in *DNAH11*. The positions of mutations are indicated in the genomic structure of *DNAH11*. The amino acid changes were compared among eight mammalian species by conservation analysis. MT9: c.5470dupC (p.S1823fs); MT10: c.T8275C (p.F2759L); MT11: c.C13183T (p.R4395X).

In family 5, patient #5707, diagnosed with CHD, heterotaxy and CD, had three variants in *DNAH11*, one frameshift variant (c.5470dupC:p.S1823fs), one missense variant (c.T8275C:p.F2759L) and one stop-gain variant (C13183T:p.R4395X). We did not obtain a blood sample from the patient’s parents, so we only confirmed these variants in the DNA sample of patient #5707 by Sanger sequencing. One frameshift variant (c.5470dupC:p.S1823fs) and two variants (c.T8275C:p.F2759L and C13183T:p.R4395X) were validated (Fig. 5A). The variant (c.5470dupC:p.S1823fs) was a *novel* frameshift insertion located in exon 32 and affected the protein function, which was conserved among different species. The c.T8275C variant was a missense variant located in exon 50 and was presented in the ExAc (0.0003) and 1000 Genomes Project (0.000599042) databases. The p.F2759L change was predicted to be damaging by the PolyPhen2 and MutationTaster software programs and was conserved among different species. The variant (C13183T:p.R4395X) in exon 81 was a *novel* stop-gain type that influenced protein function. The p.R4395X variant was highly conserved among different species (Fig. 5B).

**Figure 6:**
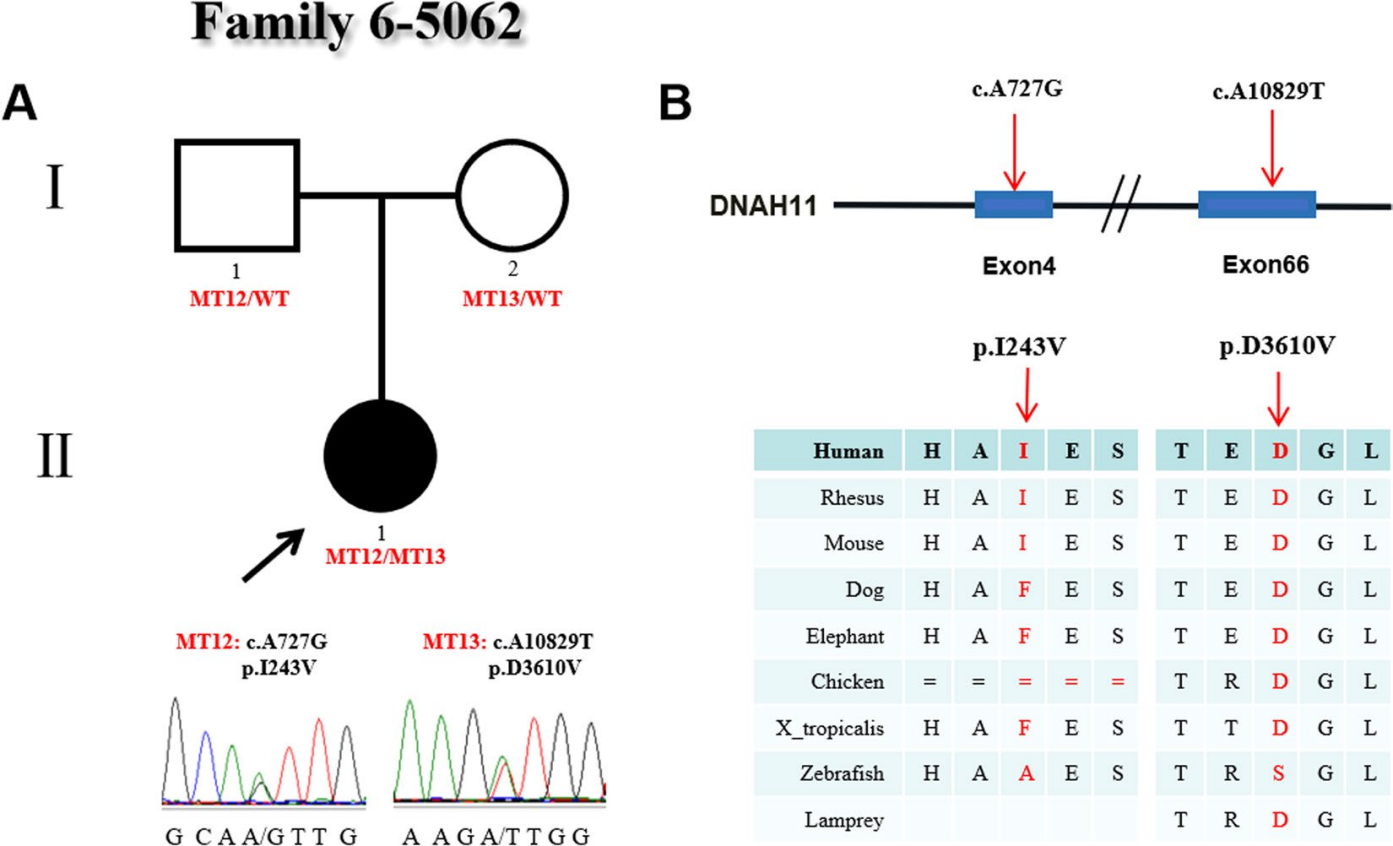
Identification of mutations in *DNAH11* in family 6. (**A**) The pedigree of family 6 (#5062). The proband carries two heterozygous mutations in *DNAH11*. Sanger sequencing confirmation is shown below the pedigrees. Locations and conservation of mutations in *DNAH11*. A black arrow indicates the proband. (**B**) Locations and conservation of mutations in *DNAH11*. The positions of mutations are indicated in the genomic structure of *DNAH11*. The amino acid changes were compared among eight mammalian species by conservation analysis. MT12: c.A727G (p.I243V); MT13: c.A10829T (p.D3610V). A square represents a male, and a circle represents a female.

In family 6 (Fig. 6A), there were three members. The proband (#5062), who carried 2 heterozygous mutations (c.A10829T:p.D3610V and c.A727G:p.I243V), was female and diagnosed with CHD and heterotaxy, including isolated right heart, PA, levo-transposition of the great arteries (L-TGA) and ASD, and showed abnormal ciliary function. His parents both showed normal phenotypes. The c.A10829T (p.D3610V) variant was a *novel* heterozygous type located in exon 66 of *DNAH11* and was inherited from the proband’s mother. Functional analysis showed the variant p.D3610V was predicted to be damaging by SIFT software and conserved among human, rhesus and dog species. The other heterozygous variant c.A727G (p.I243V) was a *novel* missense mutation type located in exon 4 of *DNAH11* and was transmitted from the proband’s father. The p.I243V change was also predicted to be damaging by SIFT software and conserved among human, rhesus and mouse species (Fig. 6B).

Based on these confirmed variants and the medical history of the families, the findings suggest that these *DNAH11* compound heterozygote variants are responsible for the development of CHD/heterotaxy syndrome.

## Discussion

Human LR asymmetry plays an important role in normal organogenesis and provides the developmental basis for correct heart looping^2^. LR asymmetry disorders in early embryonic development may result in a series of congenital birth defects, such as heterotaxy syndrome^14^. Although many genes have been reported to be associated with LR asymmetry disorders^15^, the exact aetiological mechanism of CHD and heterotaxy remain unknown. Moreover, studies have found that cilia participate in the formation of the left and right asymmetric mode by regular swinging to form a nodal flow during the embryonic period^16^. PCD and CD have been shown to be associated with CHD and heterotaxy syndrome. PCD is considered a monogenic heterogeneous recessive disorder, and CHD and heterotaxy syndrome are multiple, complex inherited diseases. There may be a link between PCD and CHD/HTX.

In this study, we found 34 potential disease-causing heterozygous variants in 11 genes present in 19 CHD/HTX patients, accounting for 45.2% of 42 CHD/HTX patients. We compared the gene mutation rate in the CHD/HTX patients with CD and CHD/HTX patients without CD and found that CHD/HTX patients with CD had a significantly higher gene mutation rate than CHD/HTX patients without CD. The results suggest that PCD-related gene mutations are significantly associated with CHD with heterotaxy and CD.

*DNAH11* is localized to the proximal region of respiratory cilia and is known as a PCD-related gene. Approximately 6% of PCDs are caused by *DNAH11* mutations^17^. Although cilia are required for LR body-axis determination and second heart field (SHF) Hedgehog (Hh) signalling, Burnicka *et al*. observed that *DNAH11* mutations did not disrupt SHF Hh signalling and caused AVSDs only concurrently with heterotaxy, a LR axis abnormality^18^.

*DNAH11* showed the highest mutation rate in this study, followed by *HYDIN* and *DNAH5*. We concluded that *DNAH11* mutations may be an important risk factor involved in the development of CHD/HTX syndrome. We reanalyzed the exome database in our previous study, including 98 CHD cases and 3 CHD/HTX cases, and found no mutation in the *DNAH11* gene among 98 CHD cases and 1 *DNAH11* mutation in 1 of 3 CHD patients with heterotaxy. We considered the 98 CHD cases as controls, fisher’s exact test revealed that *DNAH11* mutations can significantly increase the risk of developing CHD/HTX syndrome, indicating a significant association of *DNAH11* mutations with CHD and heterotaxy syndrome.

It is known that *DNAH11* mutations are inherited by autosomal recessive inheritance pattern. Bartoloni L *et al*. found a homozygous nonsense mutation (R2852X) in *DNAH11* in a patient with situs inversus totalis^19^. *DNAH11* compound heterozygotes (p.R2250*/p.Q3604*) were observed in two monochorionic biamniotic male twins with PCD^13^. Nader Nakhleh *et al*. found *DNAH11* compound heterozygotes (p.Q1507P/p.E3133K) in a patient with heterotaxy; these two mutations were predicted to be damaging and involved in hyperkinetic ciliary beats^4,20^.

*DNAH11* homozygous mutations were not observed in our study. We focused on recessive *DNAH11* mutations in the families and found six different compound heterozygous variants in six families and concluded that these compound heterozygous variants may be the main factors causing CHD/heterotaxy syndrome.

Recently, a study found that heterotaxy patients with heterozygous *DNAH6* mutations also had heterozygous mutations in *DNAH5* and *DNAHI1* genes, which experimentally showed that the trans-heterozygous interactions of *DNAH6* with *DNAI1* or *DNAH5* may contribute to heterotaxy syndrome^21^.

In our study, in addition to these *DNAH11* compound heterozygotes, we also found that the patients with the *DNAH11* heterozygous mutations possessed other heterozygous mutations in known PCD genes, such as *HYDIN* and *DNAH5*. Patients 5033, who possessed two different heterozygous variants in the *DNAH11* and *HYDIN* genes (Table 2), presented primary CD and heterotaxy/CHD, indicating that interactions between trans-heterozygous variants of *DNAH11* and *HYDIN* may be involved in heterotaxy/CHD and PCD.

Although over 40 PCD pathogenic genes were revealed, our study found compound heterozygous variants in only *DNAH11* among the CHD patients with heterotaxy, which is subject to the limited number of patients recruited in this study. We speculated that larger studies may uncover comprehensive genetic pathogenic factors related to cilia among these patient populations. However, we can still conclude that pathogenic *DNAH11* mutations are an important cause of heterotaxy with CHD. Additionally, we did not conduct deeper functional studies of these *DNAH11* heterozygous variants for pathogenic confirmation and further interpretation of the genotypic and phenotypic mechanisms, which should be the key aspects of future works.

## Conclusion

In summary, we performed targeted sequencing and Sanger sequencing to analyze the exonic regions of 37 candidate genes in 42 HTX patients with CHD from unrelated families and found 34 potential disease-causing heterozygous variants in 11 genes among the 19 CHD patients with heterotaxy syndrome. The association of *DNAH11* variants with CHD and heterotaxy was significant (*P* = 0.0001). In families, six different compound heterozygous variants of *DNAH11* were validated in family 1-5031 (p.W802X/p.M282I), family 2-5045 (p.T3460K/p.G4425S), family 3-5065 (p.G447R/p.L1157R), family 4-5130 (p.I2262T/p.D3800H), family 5-5707 (p.S1823fs/p.F2759L/p.R4395X) and family 6-5062 (p.D3610V/p.I243V).

These findings expand the spectrum of *DNAH11* gene mutations causing the development of HTX/CHD and provide an important clue for understanding the genetic mechanism of HTX/CHD syndrome.

## Methods

### Patient cohorts

Blood samples from patients or their family members were collected in accordance with the Declaration of Helsinki, and the Ethics Committees of Children’s Hospital of Fudan University (CHFU) approved this study. Written informed consent was obtained from the parents and guardians of all the probands. Informed consent has been obtained for the blood samples taken from the available family members.

Forty-two participants with CHD and heterotaxy syndrome were recruited from the CHFU, Shanghai, China, including 28 males and 14 females, with ages ranging from 0.1 to 12.1 years. Family medical history was obtained at the cardiovascular centre of the CHFU, and medical records were reviewed to confirm the disease diagnosis. Blood samples from probands and their available family members were obtained for further genetic analysis.

### Nasal tissue sampling and ciliary motion analysis

Nasal tissues were collected from patients with Rhino-Probe (Arlington Scientific, Springville, UT) curettage of the inferior nasal turbinate. Exclusion criteria included severe bleeding diathesis or conditions such as haemophilia or hereditary haemorrhagic telangiectasia syndrome. The nasal tissues were suspended in L-15 medium (Invitrogen, CA) for videomicroscopy using a Leica inverted microscope (DMIRE2) with a 67× oil objective under differential interference contrast optics. Movies were recorded at 200 frames/s at room temperature using a 680 PROSILICA GE camera (Allied Vision, PA), and digital recordings were evaluated by a blinded panel of coinvestigators. Abnormal ciliary motion was described as follows: immotile (I), discordance (D), wave (W), restricted (R) and no cilia (None).

### Blood DNA extraction

A QIAamp DNA Mini Kit (Qiagen, Hilden, Germany) was used to extract blood genomic DNA from probands and available family members according to the manufacturer’s instructions. The concentration and purity of genomic DNA were measured by absorbance at 260 and 280 nm by using a NanoDropTM 1000 Spectrophotometer (Thermo Scientific, Wilmington, USA). The extracted genomic DNA were stored at −80 °C until the samples were ready for further analysis.

### Targeted sequencing analysis of gene variants

Targeted sequencing analysis was performed for the 42 probands from the CHFU. We focused on 37 candidate PCD-related genes, namely, *ABCC4*, *ARMC4*, *C21orf59*, *CCDC39*, *CCDC40*, *CCDC65*, *CCDC114*, *CCDC151*, *CCNO*, *DNAAF1*, *DNAAF2*, *DNAFF3*, *DNAAF5*, *DNAH5*, *DNAH8*, *DNAH11*, *DNAI1*, *DNAI2*, *DNAL1*, *DRC1*, *DYX1C1*, *HEATR2*, *HYDIN*, *LRRC6*, *NAT10*, *NME8*, *PTGES*, *PTGES2*, *PTGES3*, *PTGER4*, *PTGS1*, *PTGS2*, *RSPH1*, *RSPH4A*, *RSPH9*, *SPAG1* and *ZMYND10*, intending to find novel or rare coding variants present in patients with CHD and heterotaxy syndrome.

Primers covering all exons and at least 10 bp of all intron/splice sites of these genes were designed online (https://www.ampliseq.com/). The libraries were constructed using the Ion AmpliSeq Library Kit v2.0 (Life Technologies, USA) according to the protocol. The concentration of each library was confirmed by a TaqMan Quantification Kit (Life Technologies). The Ion OneTouch 2 system with an Ion PGM Template OT2 200 Kit (Life Technologies, USA) was used to amplify pooled barcode libraries, and ion sphere particles (ISP) were enriched according to the E/S module protocol. The enriched template-positive ISPs were loaded and sequenced on an Ion 316™ Chip by PGM (Life Technologies, USA).

For each subject, base calls were detected with Torrent Suite software. Raw sequencing data were aligned against the human reference genome GRCh37/hg19 (NCBI) using NextGENe software. Single-nucleotide variations (SNVs) were aligned based on the following criteria: 1) the variant was detected on both strands of the sequence reads; 2) the minimum coverage of reads was no less than 10×; 3) the variant reads represented more than 20% of the sequence reads.

The variants filtered from NextGENe software were confirmed by Sanger sequencing and compared with 1000 Genomes (http://www.1000genomes.org) and ExAc databases (http://exac.broadinstitute.org/) as well as our laboratory’s internal databases. Additionally, the risk of SNVs was predicted using the silico tools SIFT (http://sift.jcvi.org/), PolyPhen2 (http://genetics.bwh.harvard.edu/pph2/) and MutationTaster (http://www.mutationtaster.org/). The amino acid changes were compared among eight mammalian species by conservation analysis (UCSC Genome Browser hg19).

### Statistical analysis

Statistical analysis was performed using the Chi-square test with GraphPad Prism 6.0 Software. Fisher’s exact test was used to analyze the association of *DNAH11* mutations with CHD and heterotaxy. Values were considered significant at *P* < 0.05.

## Data Availability

The datasets generated during and/or analysed in this study are available from the corresponding author on reasonable request.
